# A Systematic Review and Meta-Analysis of Molecular Characteristics on Colistin Resistance of *Acinetobacter baumannii*

**DOI:** 10.3390/diagnostics14222599

**Published:** 2024-11-19

**Authors:** Ihsan Hakki Ciftci, Elmas Pinar Kahraman Kilbas, Imdat Kilbas

**Affiliations:** 1 Department of Medical Microbiology, Faculty of Medicine, Sakarya University, 54100 Sakarya, Turkey; 2 Department of Medical Laboratory Techniques, Health Services Vocational School, Fenerbahce University, 34758 Istanbul, Turkey; elmspnrkk@gmail.com; 3 Medical Microbiology Doctorate Program, Institute of Health Sciences, Istanbul University, 34093 Istanbul, Turkey; imdtklbs@gmail.com

**Keywords:** *Acinetobacter baumannii*, colistin resistance, mcr, pmr, oxa

## Abstract

Background: This study aimed to determine the molecular epidemiology of colistin-resistant *A. baumannii* in the last ten years and the frequency of gene regions related to pathogenesis, to compare the methods used to detect genes, and to confirm colistin resistance. Methods: This meta-analysis study was conducted under Preferred Reporting Items for Systematic Reviews and Meta-Analysis Guidelines. In the meta-analysis, research articles published in English and Turkish in electronic databases between January 2012 and November 2023 were examined. International Business Machines (IBM) Statistical Package for the Social Sciences (SPSS) Statistics for Macbook (Version 25.0. Armonk, NY, USA) was used for statistical analysis. The Comprehensive Meta-Analysis (CMA) (Version 3.0. Biostat, NJ, USA) program was used for heterogeneity assessment in the articles included in the meta-analysis. Results: After evaluating the studies according to the elimination criteria, 18 original articles were included. Among colistin-resistant strains, *bla*_OXA-51_ positivity was 243 (19.61%), *bla*_OXA-23_ was 113 (9.12%), *bla*_OXA-58_ was 7 (0.56%), *bla*_OXA-143_ was 15 (1.21%), and *bla*_OXA-72_ was seen in two (0.16%) strains. The positivity rates of *pmrA*, *pmrB*, and *pmrC* were found to be 22 (1.77%), 26 (2.09%), and 6 (0.48%). The *mcr-1* rate was found to be 91 (7.34%), the *mcr-2* rate was 78 (6.29%), and the *mcr-3* rate was 82 (6.61%). Conclusions: The colistin resistance rate in our study was found to be high. However, only some research articles report and/or investigate more than one resistance gene together. Additionally, it may be challenging to explain colistin resistance solely by expressing resistance genes without discussing accompanying components such as efflux pumps, virulence factors, etc.

## 1. Introduction

*Acinetobacter baumannii* causes critical hospital-acquired infections such as respiratory tract, urinary tract, blood circulation, surgical site, and wound infections. Antibiotic misuse have led to the spread of drug-resistant strains of this microorganism, leading to the use of last resort antibiotics, such as colistin, in the treatment of these infections [1].

The clinical use of colistin began in the 1950s. Due to its toxic effects on the kidney and nervous system, it could only be used in the treatment of lung infections caused by multidrug-resistant (MDR) Gram-negative bacteria in patients with cystic fibrosis [2,3]. Today, colistin is widely used in animal feed production and carries a major health risk [4]. Following MDR, the emergence of extensively drug-resistant (XDR) and pan-drug-resistant (PDR) Gram-negative bacteria has led to the repurposing of antibiotics such as colistin, which are considered a last resort in treatment [5].

Colistin has bactericidal activity against many Gram-negative bacteria due to its interaction with the negatively charged lipid A moiety of lipopolysaccharide (LPS) [6]. Colistin has been used for some time as an antibiotic of last resort against carbapenem-resistant *A. baumannii* strains; however, according to recent reports, colistin-resistant *A. baumannii* strains appear to be increasing in clinical settings [4]. The most prominent colistin resistance mechanisms in *A. baumannii* occur due to LPS modification mediated by the PmrA/PmrB system and the loss of LPS due to impaired synthesis of lipid A [7].

LPS modification is a common mechanism of acquired colistin resistance in Gram-negative bacilli. In *A. baumannii*, phosphoethanolamine (PetN) is added to the 40-phosphate or 1-phosphate group of lipid A, reducing the negative charge of LPS and the binding affinity of colistin [8]. This type of colistin resistance occurs predominantly due to mutations in the genes encoding PmrAB, which leads to the overexpression of the PetN transferase PmrC [9].

Recently, it has been demonstrated that colistin resistance genes transferred via plasmid also have an effect on colistin resistance [10]. The *mcr-1* is a plasmid-mediated gene encoding a PetN transferase that causes colistin resistance [5]. *mcr-1* was detected for the first time in *Escherichia coli* obtained from various clinical, animal, and environmental samples in China in 2016 [11].

More than 100 variants of the *mcr* gene family (*mcr-1*-10) have been identified in bacteria isolated from animals, food, humans, and the environment [3,12]. The *mcr-1* and *mcr-4.3* variants are the most frequently detected *mcr* gene regions in *A. baumannii*.

Nosocomial infections caused by multidrug-resistant (MDR) bacteria cause increased mortality, morbidity, and treatment costs and continue to endanger the lives of patients in hospitals, especially those with compromised immune systems [13]. Therefore, the identification of resistance genes and other resistance mechanisms in *A. baumannii* is of great importance for the control of hospital infections caused by MDR bacteria. These genetic analyses can provide critical data for the development of treatment strategies and the prevention of the spread of resistant strains.

This meta-analysis aimed to examine the molecular epidemiology of colistin-resistant *A. baumannii* in the last decade, to determine the prevalence of gene loci associated with resistance, to discuss the methods used to define related genes, and to use tests to confirm colistin resistance.

## 2. Materials and Methods

### 2.1. Literature Search and Research Strategies

This systematic review was conducted under the guidance of the Preferred Reporting Items for Systematic Reviews and Meta-Analysis Guidelines [14]. The study included research articles published in English and Turkish in the PubMed, Excerpta Medica Database (EMBASE), Scopus, Google Scholar, Web of Science, Elton B. Stephens Company (EBSCO), and Turkish Medline databases between January 2012 and November 2023.

The literature was searched for original articles in English and Turkish reporting on colistin resistance around the world, using the key terms “kolistin Acinetobacter izolatlari”, “kolistin direncli *A. baumannii* izolatları”, “kolistin direncli *A. baumannii* izolatlarının karakterizasyonu”, “kolistin direncli *A. baumannii* izolatlarının epidemiyolojisi”, “kolistin direncli *A. baumannii* virulans genleri”, “colistin-resistant *Acinetobacter baumannii*”, “colistin-resistant *Acinetobacter baumannii* virulence genes” and “Molecular characterization of colistin-resistant *Acinetobacter baumannii*”. The screening and collection of articles containing the keywords were performed by three different authors. The authors independently evaluated the research articles that met the inclusion criteria. Discrepancies that emerged were discussed in a scientific meeting organized by the team and a consensus was reached.

### 2.2. Inclusion and Exclusion Criteria

Studies containing more than ten samples of *A. baumannii* strains isolated from clinical specimens, original studies, studies published between January 2012 and November 2023, and studies whose publication languages were English and Turkish were included.

Book chapters, systematic reviews, compilations, meta-analysis studies, case reports, letters to the editor, studies whose full text was not available, studies that did not identify the strains used at the species level, studies with fewer than ten strains included, articles in languages other than English and Turkish, congress proceedings, animal studies, data-inconsistent studies, and repetitive studies were eliminated.

### 2.3. Data Collection and Quality Assessment

During the literature search phase, titles and abstracts were reviewed and the full texts of studies deemed appropriate by consensus of the authors were obtained. Spreadsheets were prepared, using Microsoft Excel, for data collection. In these spreadsheets, information such as the author information for each study, the year the study was published, the institution where the research was conducted, sample size, the number of confirmed samples, the methods applied, the methods used for detecting colistin resistance, and relevant gene regions were recorded.

Three authors assessed the studies’ quality using the prevalence studies checklist from the Joanna Briggs Institute guidelines. The checklist consists of nine questions that reviewers answer for each survey. A “Yes” answer to each question received 1 point. Thus, the final score of each study ranged from 0 to 9. A score of 4 to 6 was considered medium-quality, and a study scoring 7 to 9 was regarded as high-quality [15].

### 2.4. Statistical Analysis

Statistical analyses were performed using IBM SPSS Statistics for Macbook (Version 25.0. Armonk, NY, USA) package program. A one-way analysis of variance (ANOVA) test was used to analyze whether the colistin resistance rate differed statistically over time. The statistical significance level was *p* < 0.05. Differences between groups were analyzed to be significant using the Tukey test, one of the post hoc analyses.

Forest plots and statistical tests (I^2^ heterogeneity measurement) were used to evaluate heterogeneity in the articles included in the meta-analysis. The design type (fixed or random effects model) was determined based on the test results. These tests were performed in the CMA (Version 3.0. Biostat, NJ, USA) program.

## 3. Results

**Figure 1:**
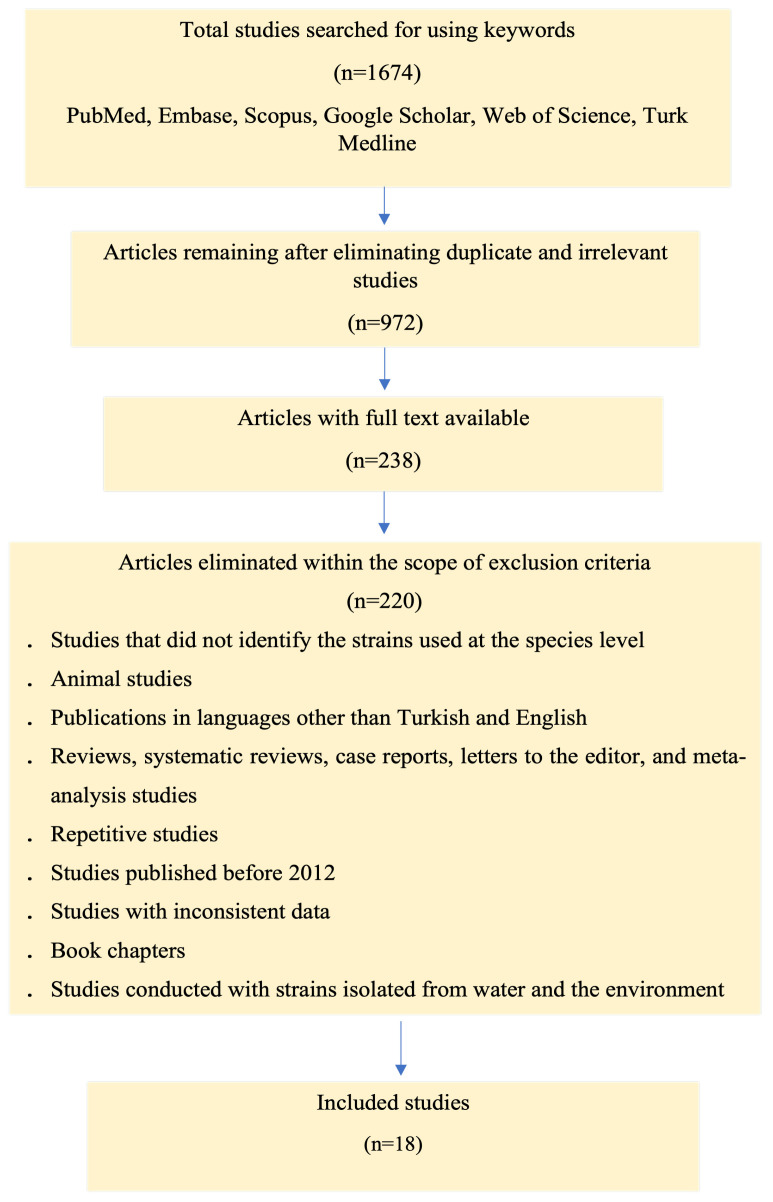
PRISMA flow diagram illustrating the literature search and study selection process.

A total of 1674 studies published between 2012 and 2023 were identified as a result of scanning the databases. As a result of evaluating these studies according to the inclusion and exclusion criteria, it was determined that 18 original articles met the appropriate conditions (Figure 1). As a result of the quality evaluation of the studies, it was determined that seven (38.88%) were of medium quality and eleven (61.12%) were of high quality.

The included publications were analyzed and divided into three periods: 2012–2019 (*n* = 7), 2019–2020 (*n* = 6), and 2021–2023 (*n* = 5). According to Begg’s rank correlation analysis, no evidence of publication bias was detected (*p* = 0.236; *p* > 0.05).

It was determined that the most frequently used identification method was Polymerase Chain Reaction (PCR), which was used in 17 of 18 studies. It was determined that the most commonly used methods for identification purposes other than PCR were sequencing in four of the 18 studies and Matrix Assisted Laser Desorption Ionization-Time of Flight Mass Spectrometry (MALDI-TOF MS) in four. 

It was determined that the most frequently used method for determining antibiotic resistance/susceptibility rates was the VITEK-2 system (bioMerieux, Marcy l’Etoile, France). It was also determined that the most commonly used identification method was the VITEK-2 system, followed by the microdilution method (four studies) and Kirby–Bauer disk diffusion methods (four studies). In 2017, Clinical & Laboratory Standards Institute (CLSI) and European Committee on Antimicrobial Susceptibility Testing (EUCAST) established the broth microdilution method as the reference method for determining colistin susceptibility, in accordance with International Organization for Standardization (ISO) 20776 [16]. Disk diffusion and gradient strip methods are not recommended due to their limited ability to diffuse in agar [17]. It has been reported that the MicroScan test system, which works with the broth microdilution principle, has high performance when detecting colistin resistance, with a very large error rate of 0.8% [18]. MALDI-TOF MS rapidly detects antibiotic resistance in bacteria through protein analysis (sensitivity: 91% and above) [19]. VITEK-2 is an automated system that determines the susceptibility of bacterial cultures by placing various antibiotic concentrations on cards. The sensitivity rate of the VITEK-2 system in detecting colistin resistance in *A. baumannii* has been reported to be 90.1% [20]. Although the detection of mcr genes through PCR has been reported to have sensitivity and specificity rates close to 100%, it is not sufficient for rapid diagnosis of the *mcr-1* gene in *A. baumannii*; therefore, it is recommended that PCR-positive samples be confirmed using DNA sequencing [21]. Sequencing provides high accuracy by directly analyzing genetic material, though it is an expensive and time-consuming method [22].

While it was determined that there were differences between the methods used to determine colistin resistance/sensitivity ratios, the most frequently used method was the microdilution method. It was determined that agar dilution and gradient strip test were the most commonly used methods, after the microdilution method, for confirming colistin resistance. It was observed that the included studies were frequently conducted in Middle Eastern countries and that most reports were from Iran and Egypt. Information regarding all the studies is given in Table 1.

**Figure 2:**
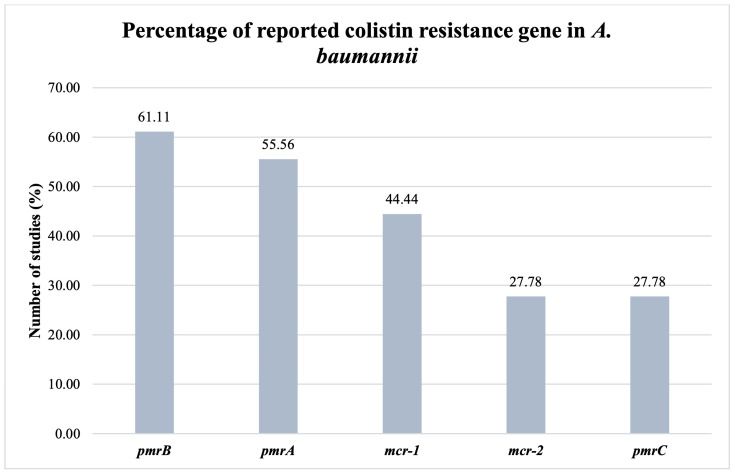
Percentage of studies reporting colistin resistance genes in *A. baumannii*.

It was determined that 58.22% of all patients in the articles included in the study were male, and 41.78% were female. A total of 1239 strains were studied from all studies, and 357 (28.81%) were found to be resistant to colistin. The average colistin resistance rate was determined to be 39.1 ± 32.6%. It was found that there was no statistically significant difference in colistin resistance rates over the years (*p* = 0.28; *p* > 0.05). In research articles with a sample size of less than 50, the average rate of colistin resistance in *A. baumannii* isolates was 58.30% (95% CI: 5–100%), while in studies with a sample size of 50 or more, it was calculated to be 23.74% (95% CI: 2.14–76.03%) in studies. In studies in African countries, the colistin resistance rate was, on average, 37.41% (95% CI: 9.38–76.92%). In studies in Asian countries, it was 32.73% (95% CI: 2.14–100%); in European countries, it was 52.86% (95% CI: 28.57–100%) on average (Figure 2).

Among colistin-resistant strains, *bla*_OXA-51_ positivity was 243 (19.61%), *bla*_OXA-23_ 113 (9.12%), *bla*_OXA-58_ 7 (0.56%), *bla*_OXA-143_ 15 (1.21%), *bla*_OXA-72_ was seen in two (0.16%) strains, while *bla*_OXA-24_ was tested in three of the included studies and no positive strain was detected. 

When the results of the studies included in our meta-analysis were examined, the positivity rates of *pmrA*, *pmrB*, and *pmrC* were found to be 22 (1.77%), 26 (2.09%), and 6 (0.48%), respectively. The *mcr-1* rate was found to be 91 (7.34%), the *mcr-2* rate was 78 (6.29%), and the *mcr-3* rate was 82 (6.61%), while *mcr-4* was tested in two studies and *mcr-5* was tested in three studies, and no positivity was detected. *mcr-1*+*mcr-2*, *mcr-1*+*mcr-3*, and *mcr-1*+*mcr-2*+*mcr-3* genes were detected in a single study and positivity rates were reported as 61.15% (74), 63.63% (77), and 54.54% (66), respectively (Table 2).

It was observed that *bap*, *ompA*, *bla*_PER_, integron *intI2*, integron *intI3*, and *bla*_NDM_ genes were tested in one study each, and the positivity rates were reported, respectively, as 2.85% (2), 2.85% (2), 2.85% (2), 64.46% (78), 66.94% (81), and 5.20% (5). 

Colistin-resistant *A. baumannii* strains were isolated from intensive care wards in four (22.22%) studies and from 14 patients in various inpatient wards (77.78%). No statistically significant difference was detected in the rate of colistin resistance in terms of clinical diversity (*p* = 0.89; *p* > 0.05).

Ten studies used only PCR to detect gene regions, three used PCR + sequencing, three used PCR + MALDITOF MS, one used only sequencing, and one used only MALDI-TOF MS. The most commonly used method for detecting antibiotic resistance was VITEK-2 (10 studies); the other methods are shown in Table 1. It was determined that the prevalence rates of *bla*_OXA-51_, *bla*_OXA-23_, *pmrA*, and *pmrB* genes did not show a statistically significant difference according to the gene region detection method (*p* = 0.69; *p* = 0.47; *p* = 0.36; *p* = 0.44, *p* > 0.05).

Four studies used *A. baumannii* ATCC 19606 as a positive control, two studies used *mcr-1* positive *E. coli*, one study used *E. coli* NCTC 1384, and one used *A. baumannii* ATCC BAA-747. The other studies did not report the positive controls they used. Twelve included studies used the Clinical & Laboratory Standards Institute (CLSI) as a guideline, while four used the European Committee on Antimicrobial Susceptibility Testing (EUCAST). Two of the studies used both CLSI and EUCAST as guidelines. The use of different guidelines for antibiotic susceptibility testing may lead to variations in breakpoints, test methods, and susceptibility categories. Since the CLSI- and EUCAST-recommended breakpoints for colistin differ, different susceptibility results may have occurred for the same isolate. This may have led to bacteria being considered susceptible in one guideline and being classified as resistant in another.

**Figure 3:**
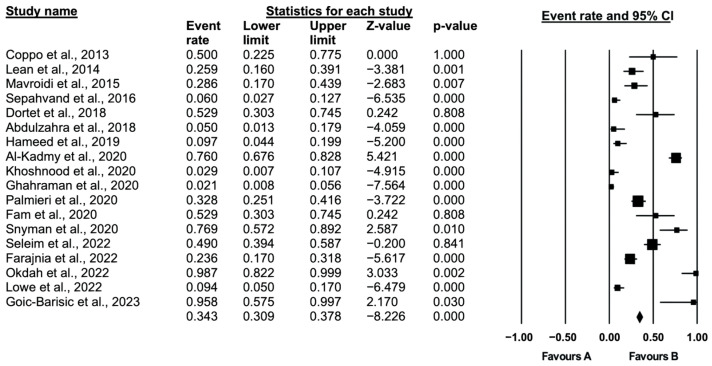
Forest plot of studies [6,10,23,24,25,26,27,28,29,30,31,32,33,34,35,36,37,38].

The studies on heterogeneity levels (i^2^) were divided into groups, as shown in Table 2. According to Cooper, Hedges and Valentin (2009), an i^2^ value of over 75% indicates a high level of heterogeneity [39]. This result required estimating the actual effect size with a random effects model. The effect size calculated with the random effects model in the study and the weights of the studies in the meta-analysis are shown as a forest plot graphic in Figure 3. From the included studies, the pooled prevalence of colistin resistance in *A. baumannii* clinical isolates was 34% (95% confidence interval (CI): 30–47%), ranging from 0.2% to 98% (Figure 3). The symmetric funnel plot showed no evidence of publication bias.

## 4. Discussion

*A. baumannii* is an important bacterium that causes a variety of healthcare-associated infections, particularly in immunocompromised patients [40]. These patient groups include patients in the intensive care unit, patients requiring long-term care, patients undergoing surgery, patients undergoing central vascular catheterization, patients undergoing tracheostomy, patients experiencing enteral bleeding, and low-birth-weight neonates [41,42]. Although mortality rates due to *A. baumannii* infections vary between 12 and 50%, these rates can vary greatly depending on factors such as patients’ accompanying health problems, length of hospital stay, demographic factors, and the sensitivity of the strains causing the infection to antibiotics [43,44].

*A. baumannii* drug resistance spreads quickly worldwide and is affected by various factors. The most important of these factors is the misuse and excessive use of antibiotics. Increasing resistance to first and second-line antibiotics has made it necessary to use colistin as a last-resort treatment. Since using colistin in the treatment of various infections, the number of studies reporting colistin-resistant *A. baumannii* strains has increased rapidly [4,45]. Therefore, in our meta-analysis study, the prevalence of antibiotic resistance genes in colistin-resistant *A. baumannii* strains was investigated, and data on gene regions that play a role in the expression of colistin resistance in these strains, as well as other resistance mechanisms accompanying colistin resistance, were presented.

According to the results of our study, the average colistin resistance rate in the 18 included articles was 39.1%. Mohammadi et al. [46] (2017) included 448 *A. baumannii* strains in their meta-analysis study and reported the colistin resistance rate as 2%. In Turkey, Ciftci et al. [47] (2022) included studies published between 2005 and 2020 in their meta-analysis and reported the colistin resistance rate as 7.9%. The study’s data show that colistin resistance was reported as 2.94% between 2011 and 2015, but this rate changed to 13.42% between 2016 and 2020. In recent studies, colistin resistance rates from India, Pakistan, Turkey, and Sweden have been reported as 10.1%, 9.6%, 28%, and 36.36%, respectively [10,48,49,50]. Our meta-analysis findings show that the colistin resistance rate in *A. baumannii* strains is higher compared to these studies. This rate was found to be highest in studies conducted in the European continent (52.85%). Differences in colistin resistance between countries may vary depending on antibiotic use habits, health infrastructure, infection control policies, and genetic characteristics. The high colistin resistance rates in Turkey can be attributed to the excessive and incorrect use of antibiotics in the food, feed, and livestock industries, as well as their use in the treatment of infectious diseases. In India and Pakistan, inadequate sanitation conditions and deficiencies in the health system contribute to the increase in colistin resistance. In developed countries such as Sweden, although colistin resistance rates remain at lower levels thanks to the strict control of antibiotic use and the effectiveness of infection control measures, bacterial transfer due to food import and tourism activities is the biggest risk factor. The patient population from which strains are isolated in studies may also affect resistance rates. For example, studies that select only carbapenem-resistant strains may show higher overall resistance rates. Therefore, the high heterogeneity in colistin resistance observed across countries and studies is likely to vary significantly depending on factors such as the clinical characteristics of the selected patient group, treatment history, and the health, food, and migration policies of the countries. 

In this meta-analysis, the prevalence of *bla*_OXA-51_ in colistin-resistant *A. baumannii* strains was 19.61% (243); *bla*_OXA-23_ was 9.12% (113), *bla*_OXA-58_ was 0.56% (7), *bla*_OXA-143_ was 1.21% (15), and *bla*_OXA_-72 was 0.16% (2). *bla*_OXA-24_ was tested in three of the included studies, and no positive strains were detected. According to the data of the systematic review conducted by Kahraman Kılbas et al. [51] (2022) on carbapenem-resistant *A. baumannii* strains in Turkey, the incidence rates of *bla*_OXA_ genes were determined to be as follows: *bla*_OXA-23_ (76.4%), *bla*_OXA-23-like_ (68.6%), *bla*_OXA-24/40_ (1.2%), *bla*_OXA-24/40-like_ (3.4%), *bla*_OXA-51_ (97.0%), *bla*_OXA-51-like_ (98.6%), *bla*_OXA-58_ (8.4%), and *bla*_OXA-58_-like (17.1%), respectively.

The recent emergence of colistin resistance and the discovery of mobile colistin resistance (*mcr*) genes encoding the phosphoethanolamine transferase enzyme that changes the cell wall have made overcoming this resistance even more difficult. *mcr* genes have been associated with plasmids passed by conjugation [52]. The global spread of *mcr* genes has been extensively documented. Three hundred fifty-seven colistin-resistant *A. baumannii* strains were isolated from the studies in our meta-analysis. *mcr-1* positivity was reported in 91 (7.34%) of these strains, *mcr-2* positivity was reported in 78 (6.29%), and *mcr-3* positivity was reported in 82 (6.61%). *mcr-4* was tested in two studies and *mcr-5* in three studies, and no positivity was detected. *mcr-1*+*mcr-2*, *mcr-1*+*mcr-3*, and *mcr-1*+*mcr-2*+*mcr-3* genes were detected in a single study, and their positivity rates were reported to be 61.15% (74), 63.63% (77), and 54.54% (66), respectively. In our meta-analysis, all *mcr* genes were studied in countries in the Asian continent. Studies conducted in the Czech Republic suggest that food imports from America and Asia can easily transport *A. baumannii* strains carrying *mcr* genes to European countries [53,54]. 

Colistin resistance in *A. baumannii* is an acquired resistance. Chromosomal mutations in the *pmrAB* biphasic system also contribute to this resistance [55]. Considering the results of the studies included in our study, the positivity rates of *pmrA*, *pmrB*, and pmrC were found to be 1.77% (22), 2.09% (26), and 0.48% (6), respectively. In their multicenter study with colistin-resistant *A. baumannii* strains, Goic-Barisic et al. [23] (2023) reported the *pmrA* and *pmrB* positivity rates as 9.09% and 9.09%, respectively. At the same time, they did not find the pmrC gene. Snyman et al. (2020) did not detect the *pmrA*, *pmrB*, or pmrC genes with colistin-resistant strains in their study [24].

Difficulties in detecting colistin resistance are frequently brought up, and it is reported that the most appropriate method is still controversial [56]. The CLSI–EUCAST joint working group recommends minimum inhibitory concentration (MIC) measurement using the broth microdilution method [57]. In the studies included in the meta-analysis, the prevalence of colistin resistance in studies using liquid microdilution, agar dilution, gradient strip, and disk diffusion methods was found to be 55.53% (min–max: 5–100%), 37.96% (min–max: 25.93–50%), 26.1% (min–max: 23.62–28.57%), 4.07% (min–max: 2.14–6%), respectively (*p* = 0.18; *p* > 0.05). The colistin resistance rate calculated by the liquid microdilution method was observed to be higher than that of other methods. However, it should be kept in mind that these prevalence differences may vary depending on the patient’s demographic and/or clinical information. Additionally, heteroresistant *A. baumannii* isolates have been reported to be identified as susceptible to colistin, leading to colistin treatment failures [58]. Therefore, since the population analysis profile (PAP) is considered the gold standard in heteroresistant strains, it is recommended that the mini-PAP method, in which the colistin MIC is determined as >2 mg/L, be included in clinical practice [59].

## 5. Conclusions

Today, low-, middle-, and high-income countries are experiencing problems due to colistin resistance. According to the data examined in this meta-analysis, it is impossible to explain colistin resistance in *A. baumannii* clinical isolates only by molecular mechanisms, because there are very few research articles reporting and/or investigating more than one resistance gene together. It may also be difficult to explain colistin resistance solely through the expression of resistance genes without discussing components such as efflux pumps, virulence factors, etc. Our study reveals common methods used worldwide to identify colistin resistance and recognized gene regions that are related to colistin resistance in *A. baumannii*. These epidemiological data will contribute to planning clinical research studies with larger samples.

*A. baumannii* has become an essential pathogen due to its frequent occurrence in healthcare-associated infections, its virulence characteristics, and the diversity of its antibiotic resistance mechanisms. Due to the increasing prevalence of MDR strains, colistin has become widely used and thus, resistance rates have increased. The diversity of colistin resistance mechanisms in *A. baumannii*, including gene transfer, requires comprehensive information on these pathways. Extensive studies are needed to shed light on potential antibiotic resistance mechanisms. Due to the increasing prevalence of colistin-resistant strains, epidemiological studies, including sequence data investigating gene regions, will contribute to re-evaluating current therapeutic approaches.

## Figures and Tables

**Table 1:** Information of the studies included in the meta-analysis [6,10,23,24,25,26,27,28,29,30,31,32,33,34,35,36,37,38].

Study	Country	Data Collection Period	Molecular Method	Genes Encoded for Colistin Resistance	Antibiotic Resistance/Identification Method	Colistin Resistance Confirmation Method	Positive Control	Guideline	Total Isolates (*n*)	Colistin Resistance Isolates *n* (%)
Seleim et al., 2022 [6]	Egypt	2011–2013	PCR, Sanger sequencing	*bla*_OXA-51_ (49), *pmrA* (4), *pmrB* (4), *mcr-1* (1)	Microdilution	Microdilution	*mcr-1*-positive *E. coli*	EUCAST	100	49 (49)
Hameed et al., 2019 [10]	Pakistan	2015–2017	PCR, Sanger sequencing	*mcr-1* (1)	Kirby–Bauer disk diffusion method	Agar dilution and microdilution	-	EUCAST	62	6 (9.68)
Goic-Barisic et al., 2023 [23]	Multicenter (Türkiye, Croatia, and Bosnia-Herzegovina)	2015	PCR	*pmrA* (1), *pmrB* (1), *mcr-1* (0), *mcr-4* (0), *mcr-5* (0)	VITEK-2, disk diffusion, and microdilution	Microdilution	-	CLSI, EUCAST	11	11 (100)
Snyman et al., 2020 [24]	South Africa	2016–2017	PCR	*pmrA* (0), *pmrB* (0), *mcr-1* (0), *mcr-4* (0), *mcr-5* (0)	VITEK-2	Microdilution and Sensitest	*E. coli* NCTC 1384	EUCAST	26	20 (76.92)
Coppo et al., 2013 [25]	Italy	2020–2021	PCR	*bla*_OXA-51_ (5), *bla*_OXA-23_ (5), *pmrA* (0), *pmrB* (1)	VITEK-2	Agar dilution	-	CLSI	10	5 (50)
Lean et al., 2014 [26]	Malaysia	2016–2018	PCR, Sanger sequencing	*bla*_OXA-51_ (14), *bla*_OXA-23_ (14), *bla*_OXA-58_ (0), *bla*_OXA-24_ (0), *pmrA* (0), *pmrB* (0)	VITEK-2	Agar dilution	-	CLSI	54	14 (25.93)
Mavroidi et al., 2015 [27]	Greece	2017–2018	PCR	*bla*_OXA-51_ (12), *bla*_OXA-23_ (7), *bla*_OXA-58_ (7)	Microscan	Gradient strip test	-	CLSI	42	12 (28.57)
Sepahvand et al., 2016 [28]	Iranian	2018–2019	PCR	*pmrA* (4), *pmrB* (3)	-	Disk diffusion and gradient strip test	-	CLSI	100	6 (6)
Dortet et al., 2018 [29]	England	2011	PCR, MALDI-TOF Ms	*bla*_OXA-23_ (8), *pmrA* (1), *pmrB* (5)	Microdilution	Microdilution	*mcr-1* positive *E. coli*	CLSI, EUCAST	17	9 (52.94)
Abdulzahra et al., 2018 [30]	Egypt	2017–2018	PCR	*bla*_OXA-51_ (2), *bla*_OXA-58_ (0), *bla*_OXA-24_ (0), *pmrA* (1), *pmrB* (2)	VITEK-2	Microdilution	-	CLSI	40	2 (5)
Al-Kadmy et al., 2020 [31]	Iraq	-	Sanger sequencing	*mcr-1* (89), *mcr-2* (78), *mcr-3* (82), *intl-2* (78), *intl-3* (81), *mcr-1*+*mcr-2* (74), *mcr-1 mcr-3* (77), *mcr-1*+*mcr-2*+*mcr-3* (66)	VITEK-2	Chromagar™ and microdilution	*A. baumannii* ATCC BAA-747	CLSI	121	92 (76.03)
Khoshnood et al., 2020 [32]	Iranian	2015–2016	PCR	*pmrA* (2), *pmrB* (2), *mcr-1* (0), *bap* (2), *ompA* (2), *bla*_PER_ (2)	VITEK-2	-	*A. baumannii* ATCC 19606	CLSI	70	2 (2.86)
Ghahraman et al., 2020 [33]	Iranian	2010	PCR	*bla*_OXA-51_ (4), *bla*_OXA-23_ (4), *mcr-1* (0)	Kirby–Bauer disk diffusion method	Kirby–Bauer disk diffusion method	*A. baumannii* ATCC 19606	EUCAST	187	4 (2.14)
Palmieri et al., 2020 [34]	Greece	2015–2016	PCR, MALDI-TOF Ms	*bla*_OXA-51_ (40)	VITEK-2	Microdilution	*A. baumannii* ATCC 19606	CLSI	122	40 (32.79)
Fam et al., 2020 [35]	Egypt	2016–2020	PCR	*bla*_OXA-51_ (9), *bla*_OXA-23_ (6), *bla*_OXA-58_ (0), *pmrA* (9), *pmrB* (4), *pmrC* (6)	VITEK-2	Microdilution	-	CLSI	17	9 (52.94)
Farajnia et al., 2022 [36]	Iranian	2018–2021	PCR	*bla*_OXA-51_ (30), *bla*_OXA-23_ (15), *bla*_OXA-58_ (0), *bla*_OXA-72_ (1), *pmrB* (2), *bla*_OXA-143_ (15), *bla*_OXA-72_ (1)	Morphological and biochemical methods	Agar dilution and gradient strip test	*A. baumannii* ATCC 19606	CLSI	127	30 (23.62)
Okdah et al., 2022 [37]	Saudi Arabia	2012–2020	MALDI-TOF Ms	*bla*_OXA-23_ (36), *bla*_OXA-72_ (1)	Microdilution	Microdilution	-	CLSI	37	37 (100)
Lowe et al., 2022 [38]	South Africa	2019–2020	PCR, MALDI-TOF Ms	*bla*_OXA-23_ (9), *mcr-1* (0), *bla*_NDM_ (5), *mcr-5* (0)	VITEK-2	Sensititre	-	CLSI	96	9 (9.38)

**Table 2:** Subgroup analysis of colistin resistance prevalence in *A. baumannii* isolates.

		Prevalence (%)	Number of Study	Point Estimate Fixed/Random	Heterogeneity (i^2^)	Publication Bias (Begg’s Test) *p*-Value	
Year	2012–2019	35.68	7	0.209/0.201	83.496	0.01	0.22
	2019–2020	36.11	6	0.462/0.296	96.151		
	2021–2023	37.55	5	0.321/0.487	92.783		
Country	Africa	37.40	3	0.263/0.325	94.374	0.01	0.39
	Asia	32.72	10	0.357/0.224	95.624		
	Europe	52.85	5	0.357/0.423	63.618		
Clinics	Intensive care	25.45	4	0.310/0.167	96.014	0.01	0.37
	Inpatient services	39.09	13	0.269/0.302	87.530		
Sample size	<50	39.87	8	0.470/0.563	84.088	0.01	0.73
	≥50	35.51	10	0319/0.173	95.565		

## Data Availability

The authors declare that all related data are available from the corresponding author upon reasonable request.
